# Association Between Endocrine Therapy and Fracture Risk in Women with Breast Cancer in Germany—A Retrospective Cohort Study

**DOI:** 10.3390/curroncol33060322

**Published:** 2026-05-29

**Authors:** Karel Kostev, Maximilian Peters, Henning Sievert, Matthias Kalder

**Affiliations:** 1 University of Marburg, Department of Gynecology and Obstetrics, University Hospital Marburg, 35043 Marburg, Germany; 2 Epidemiology, IQVIA, 60549 Frankfurt am Main, Germany; 3 Real-World Data Analytics, IQVIA, 60549 Frankfurt am Main, Germany; 4 Real-World Solutions, IQVIA, 60549 Frankfurt am Main, Germany

**Keywords:** aromatase inhibitors, tamoxifen, breast cancer, fracture, cohort study

## Abstract

Background: Aromatase inhibitors (AIs) are widely used in hormone receptor-positive breast cancer but may adversely affect bone health. Evidence on their independent association with fracture risk compared with tamoxifen (TAM) remains inconsistent. Methods: In this retrospective cohort study, women with an initial prescription of TAM or AIs between 2016 and 2024 were identified in the IQVIA Longitudinal Prescription (LRx) database. Their prescription histories were combined with records from the IQVIA Disease Analyzer (DA) database using a validated co-therapy-based approach. Patients were then followed for up to five years. Kaplan–Meier analyses estimated cumulative fracture incidence, and Cox regression models assessed associations in unadjusted, age-adjusted, and fully adjusted analyses. Results: The study included 8938 TAM users and 14,594 AI users. Five-year cumulative incidence of all fractures was higher in the AI group than in the TAM group (14.8% vs. 9.2%). In fully adjusted primary analyses, AI therapy was not significantly associated with overall fracture risk (HR 1.10, 95% CI 0.99–1.23). A modest association persisted for major osteoporotic fractures (HR 1.24, 95% CI 1.05–1.46). Secondary exploratory analyses (age-stratified and fracture-type-specific models) showed patterns consistent with the primary results but were not powered or corrected for confirmatory inference. Conclusions: Aromatase inhibitor therapy was associated with a higher fracture incidence than tamoxifen, but much of this difference was explained by age and comorbidities. Both treatment-related effects and underlying patient characteristics contribute to fracture risk, underscoring the importance of individualized bone health assessment and targeted preventive strategies in women receiving endocrine therapy.

## Simple Summary

Women with hormone receptor-positive breast cancer commonly receive aromatase inhibitors (AIs) or tamoxifen (TAM) as endocrine therapy. While AI therapy is known to reduce estrogen levels and may affect bone health, its independent effect on fracture risk compared with tamoxifen remains debated. Using real-world prescription and outpatient diagnosis data from Germany, we studied 8938 TAM users and 14,594 AI users followed for up to five years. Although crude fracture rates were substantially higher in the AI group, adjustment for age and comorbidities largely explained this difference. Only for major osteoporotic fractures did a modest independent association with AI therapy persist. These findings highlight that fracture risk in this population is shaped by both the treatment itself and underlying patient characteristics, underscoring the need for individualized bone health monitoring in women receiving endocrine therapy for breast cancer.

## 1. Introduction

Breast cancer (BC) remains the most commonly diagnosed malignancy among women worldwide, with approximately 2.3 million incident cases and 764,000 deaths globally [1], and around 72,000 new cases reported annually in Germany alone [2]. The majority of breast cancers are hormone receptor-positive (HR+), expressing estrogen and/or progesterone receptors, and account for approximately 60–80% of all BC diagnoses [3]. For these patients, endocrine therapy (ET) represents the cornerstone of systemic treatment, effectively inhibiting estrogen-driven tumor growth and thereby substantially reducing recurrence rates and breast cancer-related mortality [4,5].

Endocrine therapy is typically administered for a duration of at least five years, with the choice of agent guided by menopausal status and individual recurrence risk [6]. Tamoxifen (TAM), a selective estrogen receptor modulator, has long been a standard treatment option for both premenopausal and postmenopausal women. It exerts anti-estrogenic effects in breast tissue while displaying partial agonistic activity in other tissues, including bone and the cardiovascular system [7]. In contrast, aromatase inhibitors (AIs)—such as anastrozole, letrozole, and exemestane—suppress peripheral estrogen synthesis and have largely replaced TAM in postmenopausal patients due to superior efficacy and a more favorable risk profile with respect to thromboembolic events and endometrial cancer [7,8].

Despite their clinical benefits, both TAM and AIs are associated with adverse effects that are highly relevant for long-term survivorship. In particular, AI therapy has been consistently linked to accelerated bone loss and a potentially increased risk of fractures, reflecting profound estrogen depletion [9,10]. However, the magnitude and independence of this association remain a matter of ongoing debate. While numerous studies have reported an elevated fracture risk among AI users [9,10,11,12], emerging evidence suggests that this relationship may be substantially influenced by underlying patient characteristics and methodological factors [13,14].

Notably, a population-based cohort study from Canada observed no statistically significant difference in osteoporotic fracture risk between women treated with AIs and those receiving TAM after multivariable adjustment [15]. Similarly, an analysis based on the Manitoba registry demonstrated that, after appropriate adjustment for baseline fracture risk—including bone mineral density (BMD) and refinement of risk modeling approaches—AI therapy was not independently associated with an increased risk of major osteoporotic, hip, or overall fractures [13]. These findings suggest that previously reported excess fracture risks in AI-treated populations may partly reflect residual confounding or limitations of commonly used risk assessment tools rather than a direct causal effect of AI therapy itself.

Taken together, the existing literature highlights a complex and potentially confounded relationship between endocrine therapy and bone health outcomes in breast cancer patients. This underscores the need for large real-world analyses that carefully account for patient characteristics and treatment patterns to better understand the clinical implications of endocrine therapy on skeletal health. Therefore, the aim of the present study was to investigate the association between initial endocrine therapy (aromatase inhibitors versus tamoxifen) and the incidence of bone fractures in women with breast cancer in Germany using large real-world data.

## 2. Methods

### 2.1. Data Sources

The present retrospective cohort study was based on a combined analysis of two IQVIA databases, the Longitudinal Prescription (LRx) database and the Disease Analyzer (DA) database. The LRx database comprises approximately 80% of prescriptions reimbursed by statutory health insurance funds in Germany. Data are available at the patient level and include information on age and sex. All patient data are fully anonymized by the data provider in accordance with applicable data protection regulations. Each prescription record contains detailed product information (e.g., brand, active substance, package size, and formulation) as well as the dispensing date. The LRx database does not contain information on diagnoses or laboratory values [16].

The DA database contains anonymized data on diagnoses, prescriptions, and basic sociodemographic characteristics collected from general and specialized outpatient practices in Germany. Practices are selected based on predefined criteria, including physician age, specialty, federal state, and community size. The DA database has been shown to be representative of outpatient practices in Germany [17]. Depending on physician specialty, database coverage ranges from approximately 3% to 7%.

### 2.2. Study Population and Outcomes

This retrospective cohort study included women with an initial prescription of tamoxifen or aromatase inhibitors (anastrozole, letrozole, or exemestane) between January 2016 and December 2024 (index date) identified in the LRx database. In Germany, these medications are typically prescribed by gynecologists in the outpatient setting.

The study outcome was the proportion of women with a documented bone fracture during endocrine breast cancer therapy within up to five years after the index date. As fracture diagnoses are generally made in hospital settings and subsequently documented by general practitioners in outpatient records, fracture outcomes cannot be assessed in the LRx database.

Therefore, prescription histories from the LRx database were combined with records from the DA database using a validated co-therapy-based approach. This method identifies consistent treatment patterns across both datasets based on comparable prescription characteristics, without the use of personal identifiers. The approach is designed to operate on fully anonymized data and in accordance with applicable data protection requirements.

Each patient was followed for up to five years from the index date until the first documented fracture diagnosis in the DA database, discontinuation, switch, or end of endocrine therapy, or end of follow-up. The expected duration of each prescription was calculated based on package size, number of packages, and defined daily dose (DDD). Only records from the LRx database with treatment histories comparable to records in the DA database were included in the analysis. Patients were classified according to their initial (index) endocrine therapy prescription. In routine clinical practice, sequential endocrine therapy is common—for example, a patient may receive tamoxifen for two years followed by an aromatase inhibitor for three years. In the present analysis, patients were categorized based on the first documented prescription, and follow-up was censored at the time of documented treatment switch. Consequently, patients who switched from tamoxifen to an aromatase inhibitor (or vice versa) contributed person-time only under their initial therapy. This approach reflects an intention-to-treat-like classification by initial therapy and is a recognized limitation, as it does not capture the full complexity of sequential treatment strategies in clinical practice.

### 2.3. Statistical Analyses

Baseline characteristics were compared between women receiving tamoxifen and those receiving aromatase inhibitors using chi-square tests for categorical variables and *t*-tests for continuous variables. Kaplan–Meier analyses were used to estimate cumulative fracture incidence over time.

Three Cox proportional hazards regression models were performed to assess the association between initial therapy (aromatase inhibitors versus tamoxifen) and fracture incidence. The first model was univariable and conducted for the overall population as well as stratified by age groups (≤55, 56–65, 66–75, and >75 years). The second model was adjusted for age, and the third model was fully adjusted for age and co-diagnoses documented within 12 months prior to the index date. These included osteoporosis without fracture (ICD-10: M81), history of fractures (ICD-10: M80, S02, S12, S22, S32, S42, S52, S62, S72, S82, S92, T02, T08, T10, T12), bone metastases (ICD-10: C77.5), diabetes mellitus (ICD-10: E10–E14), hypertension (ICD-10: I10), dyslipidemia (ICD-10: E78), obesity (ICD-10: E66), depression (ICD-10: F32, F33), chronic bronchitis and chronic obstructive pulmonary disease (ICD-10: J42–J44), thyroid disorders (ICD-10: E00–E07), and rheumatoid arthritis (ICD-10: M05, M06). The selection of covariates for the fully adjusted model was based on their established or plausible association with fracture risk and their availability within the database. Specifically, conditions such as osteoporosis, prior fractures, and bone metastases are well-recognized direct determinants of skeletal fragility, while comorbidities including diabetes mellitus, chronic obstructive pulmonary disease, and rheumatoid arthritis have been associated with altered bone metabolism and increased fracture risk. Cardiometabolic conditions such as hypertension, dyslipidemia, and obesity were included as proxies of overall health status and potential contributors to fracture risk through mechanisms such as falls or reduced mobility. In addition, depression and thyroid disorders were considered due to their potential association with bone health and fracture risk, either directly or via related treatments.

We acknowledge that other relevant factors influencing fracture risk—such as bone mineral density, lifestyle factors (e.g., smoking, alcohol consumption, physical activity), and menopausal status—were not available in the database and therefore could not be included in the analyses. Consequently, the selected variables should be interpreted as a set of clinically relevant and data-available covariates rather than a comprehensive representation of all fracture risk factors, and residual confounding cannot be excluded.

Fractures were classified as major osteoporotic fractures, other osteoporotic fractures, and non-osteoporotic or undefined fractures based on ICD-10 codes (Table 1).

Kaplan–Meier curves and Cox regression analyses were additionally performed for each fracture type (major osteoporotic, other osteoporotic, and non-osteoporotic or undefined fractures).

The primary endpoints were (1) all fractures and (2) major osteoporotic fractures in the fully adjusted model. All other analyses, including age-stratified models, crude models, and other fracture categories, were considered exploratory and were not subjected to multiplicity correction. Corresponding *p*-values are reported descriptively. Analyses were conducted using SAS version 9.4 (SAS Institute, Cary, NC, USA).

## 3. Results

### 3.1. Basic Characteristics of the Study Sample

The study included 8938 women receiving tamoxifen (TAM) and 14,594 receiving aromatase inhibitors (AIs). Women treated with TAM were older than those treated with AIs (mean age: 66.7 vs. 60.2 years, *p* < 0.001), and a substantially higher proportion of TAM patients were aged ≤55 years (42.7% vs. 12.7%), whereas AI patients were more frequently represented in older age groups, particularly >75 years (32.3% vs. 16.7%). Although the mean age of the TAM group (66.7 years) was higher than that of the AI group (60.2 years), the stratified analysis by age categories reveals an apparently contradictory pattern, with a higher proportion of TAM patients in the youngest age group (≤55 years: 42.7% vs. 12.7%). This apparent inconsistency is explained by the bimodal age distribution of tamoxifen users. In clinical practice, tamoxifen is prescribed to both premenopausal women (typically younger) and older postmenopausal patients who may not tolerate AIs, while aromatase inhibitors are almost exclusively used in postmenopausal women, concentrating their distribution in middle and older age groups. The resulting bimodal distribution in the TAM group—with a large proportion of younger patients and a substantial proportion of older patients—produces a higher mean age compared with the AI group, even though the AI group has a smaller proportion in the youngest category. The full age distribution of both groups is presented in Table 2 and is consistent with established prescribing patterns in Germany.

With regard to co-diagnoses, AI-treated patients had a higher prevalence of most comorbidities, including osteoporosis (6.6% vs. 5.0%), prior fractures (7.1% vs. 4.7%), bone metastases (2.2% vs. 0.4%), diabetes (17.2% vs. 10.7%), hypertension (36.3% vs. 26.8%), dyslipidemia (19.6% vs. 14.5%), obesity (8.1% vs. 6.4%), and chronic bronchitis/COPD (6.8% vs. 5.6%) (all *p* < 0.001). No statistically significant differences were observed for depression, thyroid disorders, or rheumatoid arthritis (Table 2).

In the LRx database, the proportion of patients receiving a bisphosphonate or denosumab prescription on the index date was low in both groups (1.6% in AI-treated and 0.5% in TAM-treated patients). During follow-up and prior to fracture diagnosis, 12.5% of AI-treated and 5.2% of TAM-treated women received at least one such prescription.

### 3.2. Cumulative Incidence of Fractures

**Figure 1:**
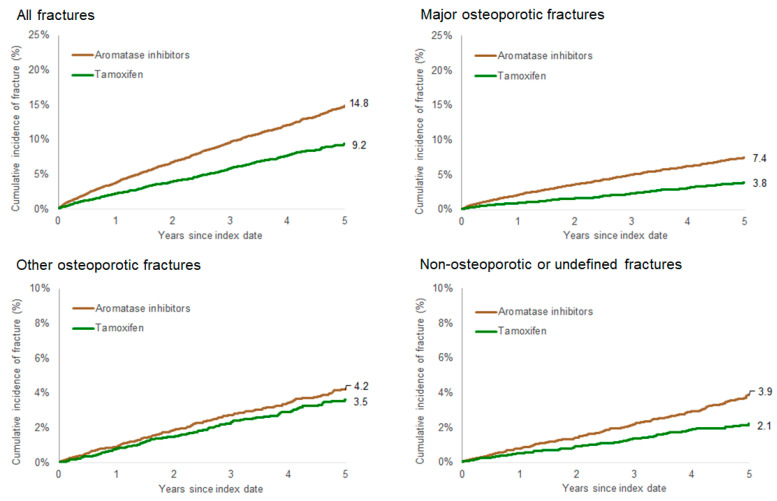
Cumulative incidence of bone fracture among women treated with TAM and AIs.

Kaplan–Meier analyses showed a consistently higher cumulative incidence of fractures among women receiving AIs compared with TAM over the entire follow-up period. After five years, the cumulative incidence of all fractures was 14.8% in the AI group and 9.2% in the TAM group (Figure 1).

When stratified by fracture type, the cumulative incidence of major osteoporotic fractures was 7.4% in AI-treated patients compared with 3.8% in TAM-treated patients. For other osteoporotic fractures, the corresponding values were 4.2% and 3.5%, respectively, while for non-osteoporotic or undefined fractures, the cumulative incidence was 3.9% in the AI group and 2.1% in the TAM group (Figure 1).

### 3.3. Association Between AI Therapy and Fracture Risk

**Figure 2:**
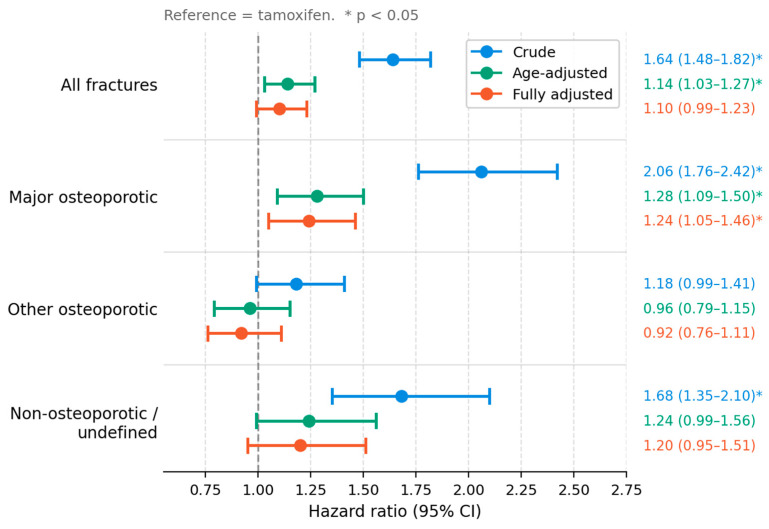
Hazard ratios (95% confidence intervals) for the association between aromatase inhibitor versus tamoxifen therapy and fracture risk by Cox regression model (crude, age-adjusted, and fully adjusted) and fracture type.

In crude Cox regression analyses, AI therapy was associated with a higher incidence of all fractures compared with TAM (HR: 1.64, 95% CI: 1.48–1.82). This association was particularly pronounced for major osteoporotic fractures (HR: 2.06, 95% CI: 1.76–2.42, *p* ≤ 0.003), while no statistically significant association was observed for other osteoporotic fractures (HR: 1.18, 95% CI: 0.99–1.41). For non-osteoporotic or undefined fractures, a higher incidence was also observed (HR: 1.68, 95% CI: 1.35–2.10). After adjustment for age, the association between AI therapy and fracture incidence remained statistically significant for all fractures (HR: 1.14, 95% CI: 1.03–1.27, *p* < 0.05) and major osteoporotic fractures (HR: 1.28, 95% CI: 1.09–1.50, *p* ≤ 0.003). In the fully adjusted model, the association with all fractures was no longer statistically significant (HR: 1.10, 95% CI: 0.99–1.23), whereas a modest association persisted for major osteoporotic fractures (HR: 1.24, 95% CI: 1.05–1.46). No statistically significant associations were observed for other osteoporotic or non-osteoporotic fractures in adjusted models (Figure 2).

**Figure 3:**
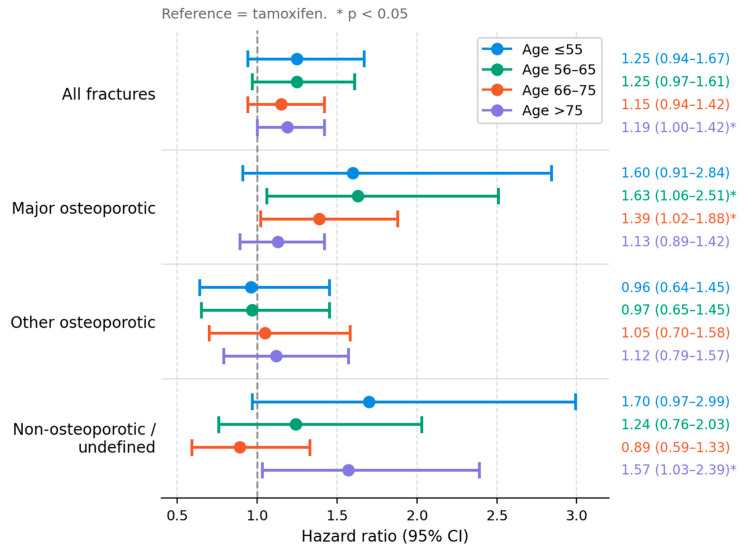
Hazard ratios (95% confidence intervals) for the association between aromatase inhibitor versus tamoxifen therapy and fracture risk stratified by age group (crude model) and fracture type.

In age-stratified analyses, associations were generally weaker and not statistically significant in younger age groups, whereas in patients aged >75 years, AI therapy was associated with a higher incidence of all fractures (HR: 1.19, 95% CI: 1.00–1.42) and non-osteoporotic fractures (HR: 1.57, 95% CI: 1.03–2.39, *p* < 0.05) (Figure 3).

## 4. Discussion

In this large retrospective cohort study based on German real-world data, the overall pattern was clear: crude fracture rates were higher among women receiving aromatase inhibitors, but these differences were substantially reduced once age and comorbidities were taken into account. For major osteoporotic fractures, we observed a small to moderate association with AI therapy in the fully adjusted model. In exploratory age-stratified analyses, the association appeared stronger in some older age groups, although these findings should be interpreted cautiously due to smaller sample sizes and their exploratory nature. Taken together, these findings indicate that both the endocrine therapy itself and the underlying patient characteristics contribute to fracture risk. While AI therapy is associated with higher fracture rates, much of this risk is shaped by age, comorbidities, and prior skeletal vulnerability.

These results should be interpreted in the context of a heterogeneous body of literature. Several randomized trials and meta-analyses have reported a higher fracture risk in women treated with AIs compared with tamoxifen. For example, in the BIG 1-98 trial, fractures were more frequent in patients receiving letrozole than in those receiving tamoxifen [18]. Likewise, the systematic review and meta-analysis by Tseng et al. [9] and the more recent meta-analysis by Qu et al. [10] both concluded that AIs are associated with a higher fracture risk. At the same time, our findings are consistent with the Canadian population-based cohort study by Blanchette et al. [15], in which the association between endocrine therapy type and osteoporotic fracture was no longer statistically significant after multivariable adjustment, as well as with the registry-based analysis by Leslie et al. [13], which showed that AI use was not independently associated with fracture risk after detailed assessment of baseline skeletal risk, including bone mineral density and FRAX-based modeling.

The discrepancy between studies is likely multifactorial and reflects methodological heterogeneity. First, studies differ substantially in outcome definitions. Some focused on all clinical fractures [11], whereas others evaluated osteoporotic fractures [12] or specific fracture types such as hip fractures [19]. In our study, fracture outcomes were analyzed both overall and by fracture type, with the strongest crude association observed for major osteoporotic fractures, while associations for other osteoporotic and non-osteoporotic fractures were weaker.

Second, the choice of comparator is highly relevant. Tamoxifen is not a neutral comparator with respect to bone health, particularly in postmenopausal women, as it may exert bone-preserving effects [14,20]. Consequently, comparisons between AIs and tamoxifen may amplify relative differences compared with studies using other reference groups.

Third, differences in confounder adjustment and data availability across studies may contribute to variability in findings. Our data clearly demonstrate the impact of baseline differences: AI-treated patients had higher prevalences of osteoporosis, prior fractures, bone metastases, diabetes, hypertension, dyslipidemia, obesity, and chronic obstructive pulmonary disease compared with tamoxifen-treated patients. After adjustment for these factors, the association with overall fractures was substantially reduced. This does not imply that previous studies were inadequately adjusted; rather, the type and granularity of available covariates differ considerably between databases. While some datasets include detailed measures such as bone mineral density, others—like ours—rely on routinely documented diagnoses and prescriptions. These differences can materially influence effect estimates and may explain why some real-world studies report stronger associations than others.

Fourth, differences in study populations are also important. In routine clinical practice, AIs are predominantly prescribed to postmenopausal women, whereas tamoxifen is used in both premenopausal and postmenopausal patients, resulting in inherently different populations. In our cohort, tamoxifen users were substantially younger, while AI users were more frequently represented in older age groups. Given that age is one of the strongest determinants of fracture risk [21,22], even moderate differences in age distribution or menopausal status may significantly influence comparative risk estimates.

Overall, our findings do not contradict the biological plausibility that AIs adversely affect bone metabolism. Bone loss under AI therapy is well documented, and reductions in bone mineral density during AI treatment have been consistently reported [23,24]. Rather, our results suggest that the translation of these biological effects into clinically observed fracture risk in routine care is strongly influenced by baseline fracture risk, patient selection, outcome definitions, and analytical strategies.

From a clinical perspective, these findings support an individualized approach to bone health in women receiving endocrine therapy. The choice of endocrine treatment should not be based solely on fracture risk; however, systematic assessment of bone health remains essential, particularly in older patients and in those with osteoporosis, prior fractures, or multiple comorbidities. Current recommendations for bone-protective management in breast cancer patients, therefore, remain highly relevant [25].

This study has several limitations. Despite adjustment for multiple co-diagnoses, residual confounding cannot be excluded. Important determinants of fracture risk—such as bone mineral density, smoking, alcohol consumption, physical activity, calcium and vitamin D status, and menopausal status—were not available in the databases. In addition, information on tumor stage was lacking, and previous or concomitant hospital-based cancer treatments, including chemotherapy, radiotherapy, ovarian suppression, or other interventions affecting bone metabolism, were not captured. Surveillance bias cannot be ruled out, as patients receiving different endocrine therapies may differ in healthcare utilization, potentially influencing the likelihood of fracture detection. Fracture outcomes were based on diagnoses recorded in routine outpatient care, which may undercapture less severe events. Mortality data were not available. We examined the prevalence of bisphosphonate or denosumab co-prescription at the index date in the LRx database. Only 1.6% of AI-treated and 0.5% of TAM-treated patients received such therapy on the index date, suggesting that bone-modifying agent use at treatment initiation was unlikely to have materially confounded the observed associations. During follow-up and prior to fracture diagnosis, however, 12.5% of AI-treated and 5.2% of TAM-treated women received at least one bisphosphonate or denosumab prescription. Due to the cohort design, time-varying bone-modifying agent use during follow-up could not be incorporated into the regression models, and we cannot fully exclude the possibility that differential use of these agents over time contributed to some attenuation of the observed association between AI therapy and fracture risk. As with all observational studies, the findings should be interpreted as associations rather than evidence of causal relationships.

The main strengths of the study are the large sample size, the use of nationwide real-world prescription data combined with outpatient diagnostic data, the follow-up of up to five years, and the possibility to compare crude, age-adjusted, and fully adjusted models. This analytical approach allowed us to quantify how strongly baseline differences between AI and tamoxifen users influenced the observed associations.

In conclusion, aromatase inhibitor therapy was associated with a higher fracture incidence than tamoxifen, but much of this difference was explained by age and comorbidities. Both treatment-related effects and underlying patient characteristics contribute to fracture risk, underscoring the importance of individualized bone health assessment and targeted preventive strategies in women receiving endocrine therapy.

## Figures and Tables

**Table 1:** Classification of fractures based on ICD-10 codes.

Fracture Category	ICD-10 Codes	Description
Major osteoporotic fractures (MOF)	M80	Osteoporosis with current pathological fracture
	S72.0–S72.2, S72.9	Hip fractures
	S22.0	Thoracic vertebra fracture
	S32.0	Lumbar vertebra fracture
	S42.2–S42.4, S42.9	Proximal humerus fractures
	S52.5, S52.9	Distal forearm fractures
Other osteoporotic fractures	S22.1–S22.5, S22.8–S22.9	Other thoracic fractures
	S32.1–S32.5, S32.7–S32.8	Pelvic and other lumbar fractures
	S82.0–S82.9	Lower leg fractures
	S92.0–S92.9	Foot fractures
Non-osteoporotic or undefined fractures	S02.0–S02.9	Skull and facial bones
	S12.0–S12.9	Cervical spine
	S42.0, S42.1, S42.7, S42.8	Clavicle, scapula, shoulder
	S52.0–S52.4, S52.6–S52.8	Other forearm fractures
	S62.0–S62.9	Wrist and hand
	S72.3–S72.4, S72.7–S72.8	Other femur fractures
	T02	Multiple fractures
	T08	Spine unspecified
	T10	Upper limb unspecified
	T12	Lower limb unspecified

**Table 2:** Basic characteristics of the study sample.

Variable	Patients Treated with TAM (%)	Patients Treated with AIs (%)	*p* Value
*n*	8938	14,594	
Age (Mean, SD)	66.7 (11.7)	60.2 (13.5)	<0.001
Age ≤ 55	3818 (42.7)	1860 (12.7)	<0.001
Age 56–65	1927 (21.6)	3835 (26.3)	
Age 66–75	1698 (19.0)	4184 (28.7)	
Age > 75	1495 (16.7)	4715 (32.3)	
Co-diagnoses			
Osteoporosis	448 (5.0)	966 (6.6)	<0.001
Bone fractures	421 (4.7)	1030 (7.1)	<0.001
Bone metastasis	36 (0.4)	324 (2.2)	<0.001
Diabetes mellitus	958 (10.7)	2516 (17.2)	<0.001
Hypertension	2395 (26.8)	5291 (36.3)	<0.001
Dyslipidemia	1297 (14.5)	2864 (19.6)	<0.001
Obesity	571 (6.4)	1175 (8.1)	<0.001
Depression	1371 (15.3)	2327 (15.9)	0.215
Chronic bronchitis and COPD	499 (5.6)	992 (6.8)	<0.001
Thyroid gland disorders	162 (1.8)	285 (2.0)	0.444
Rheumatoid arthritis	150 (1.7)	275 (1.9)	0.249

Proportions of patients given in %, unless otherwise indicated. SD: standard deviation.

## Data Availability

The data used in this study are subject to strict privacy and confidentiality requirements and therefore cannot be made publicly available. Legal restrictions prohibit the sharing of the raw data with third parties.
